# Supplementary material for: Self-Assembled Triphenylphosphonium-Conjugated Dicyanostilbene Nanoparticles and Their Fluorescence Probes for Reactive Oxygen Species
Source: Nanomaterials (Basel). 2018 Dec 12;8(12):1034. doi: 10.3390/nano8121034 (PMC6316551; doi:10.3390/nano8121034)
Supplement: Supplementary file 1 [file nanomaterials-08-01034-s001.pdf]

Supplementary Materials

Self-assembled triphenylphosphonium-conjugated dicyanostilbene nanoparticles and their fluorescence probes for reactive oxygen species

Wonjin Choi[1,↕], Na Young Lim[1,↕], Heekyoung Choi[1], Moo Lyong Seo[1,*], Junho Ahn[2,*], Jong Hwa Jung[1,*]

[1] Affiliation 1; Department of Chemistry and Research Institute of Natural Science, Gyeongsang National University, Jinju, 52828,Republic of Korea, cwj1685@gnu.ac.kr (W.C.); skdud325@gnu.ac.kr (N.Y.L.); smile377@gnu.ac.kr (H.C.); mlseo@gnu.ac.kr (M.L.S.).

[2] Affiliation 2; Composites Research Division, Korea Institute of Materials Science, 797 Changwondaero, Changwon, 51508, South Korea, junho2587@kims.re.kr (J.A.).

* Correspondence: jonghwa@gnu.ac.kr; Tel.: +82-55-772-1488

↕ These authors contributed equally to this work.

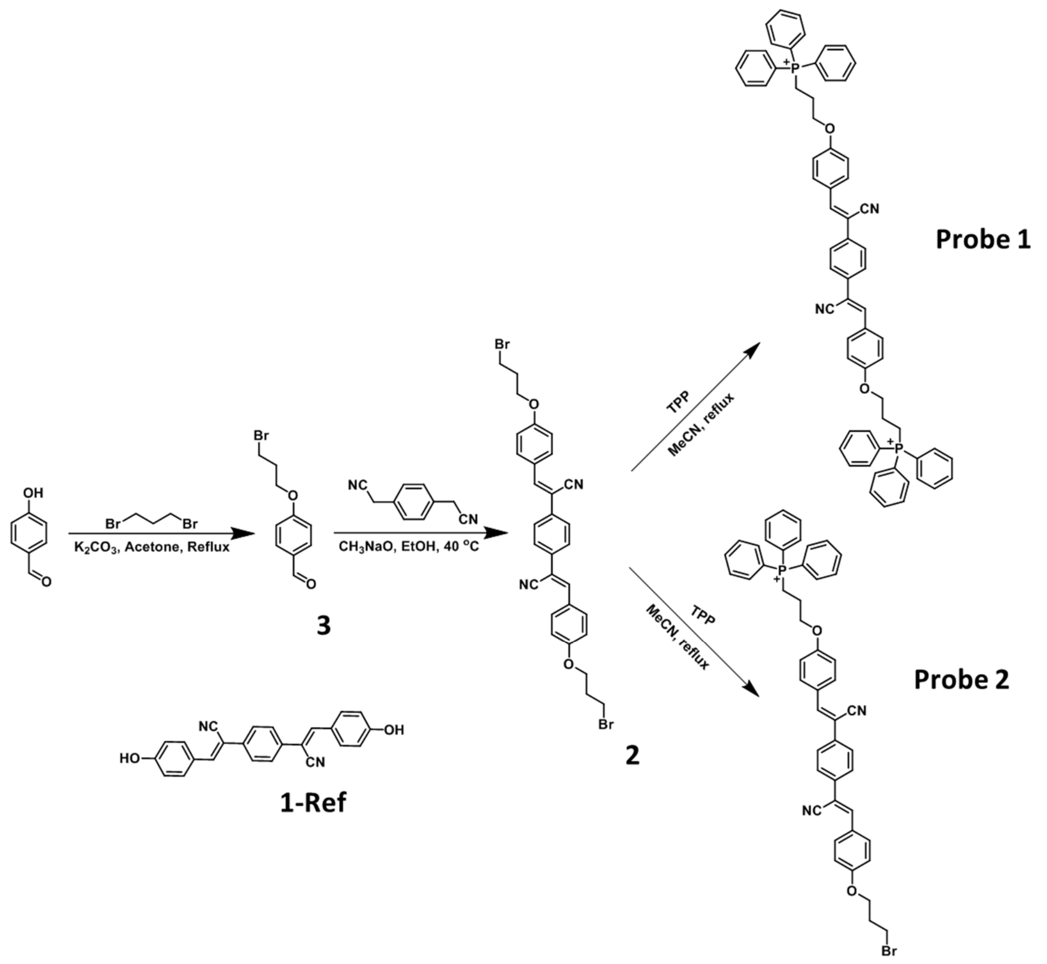

Scheme S1. Synthesis route of probes **1**, **2** and **1-Ref**.

(A)

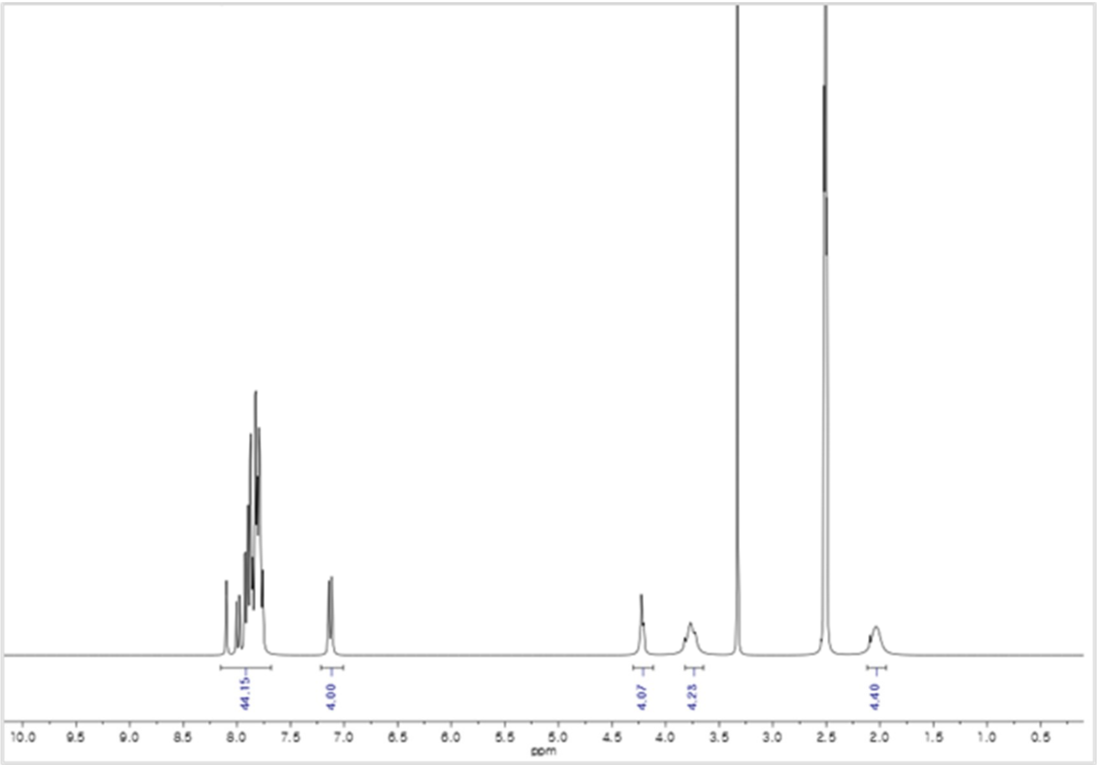
44.15
4.00
4.07
4.23
4.40
10.0 9.5 9.0 8.5 8.0 7.5 7.0 6.5 6.0 5.5 5.0 4.5 4.0 3.5 3.0 2.5 2.0 1.5 1.0 0.5
ppm

(B)

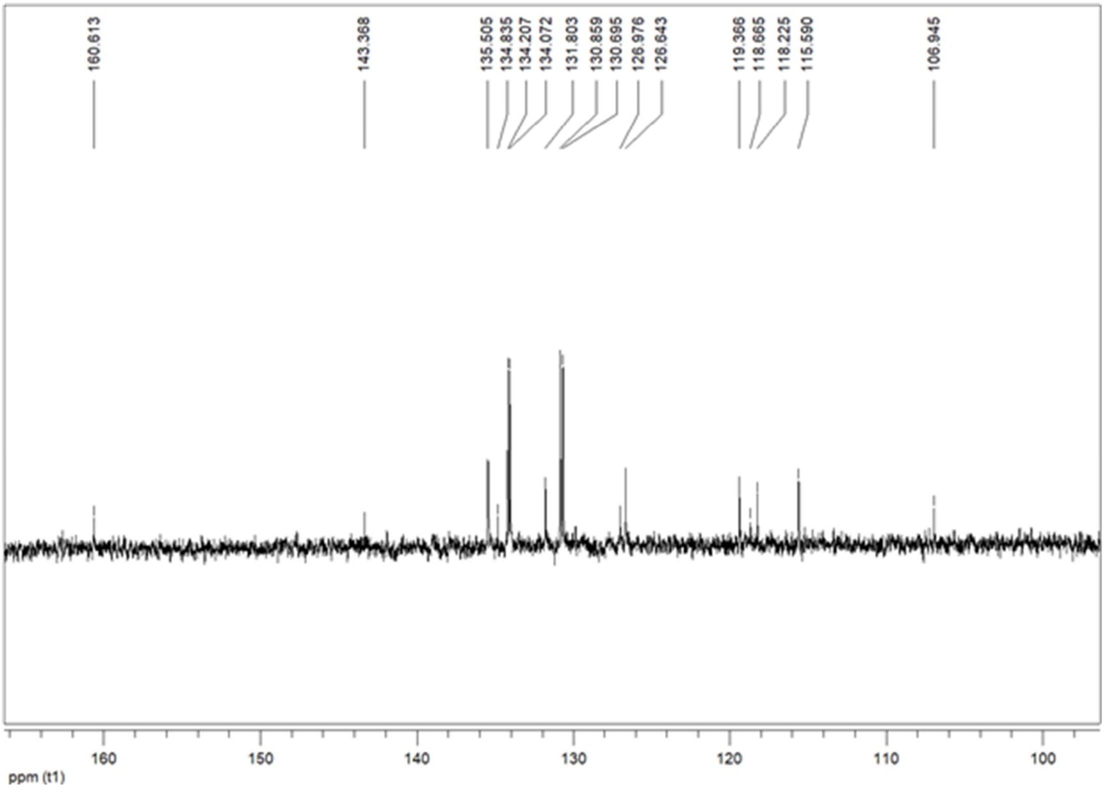
160.613
143.368
135.505
134.835
134.207
134.072
131.803
130.859
130.695
126.976
126.643
119.366
118.665
118.225
115.590
106.945
160 150 140 130 120 110 100
ppm (t1)

(C)

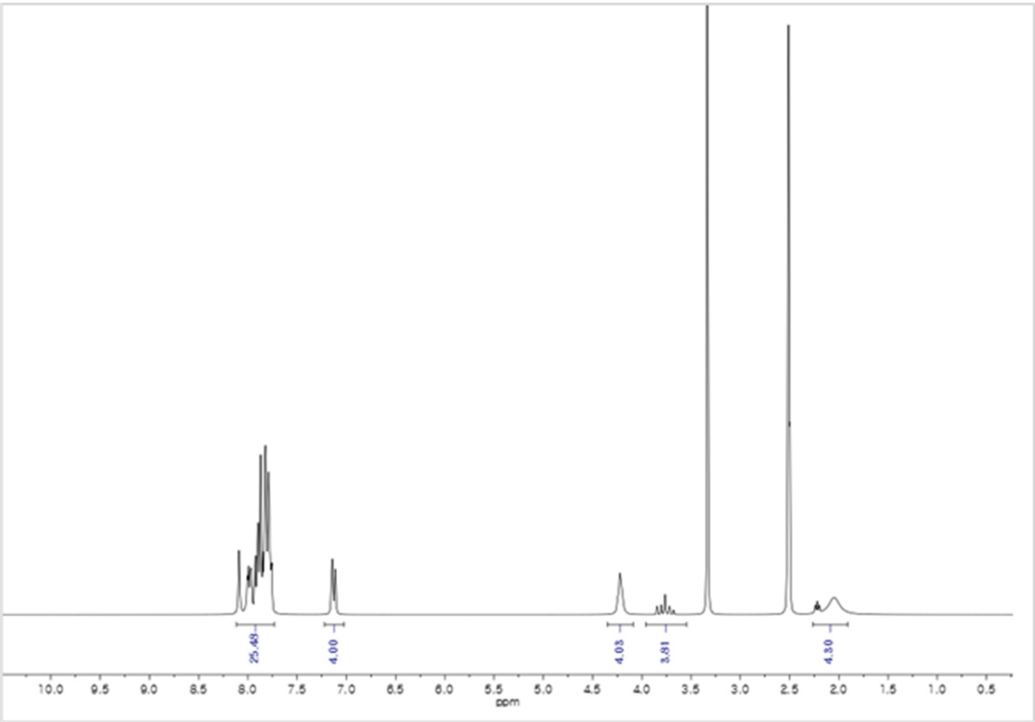
25.48
4.00
4.03
3.81
4.30
10.0 9.5 9.0 8.5 8.0 7.5 7.0 6.5 6.0 5.5 5.0 4.5 4.0 3.5 3.0 2.5 2.0 1.5 1.0 0.5
ppm

(D)

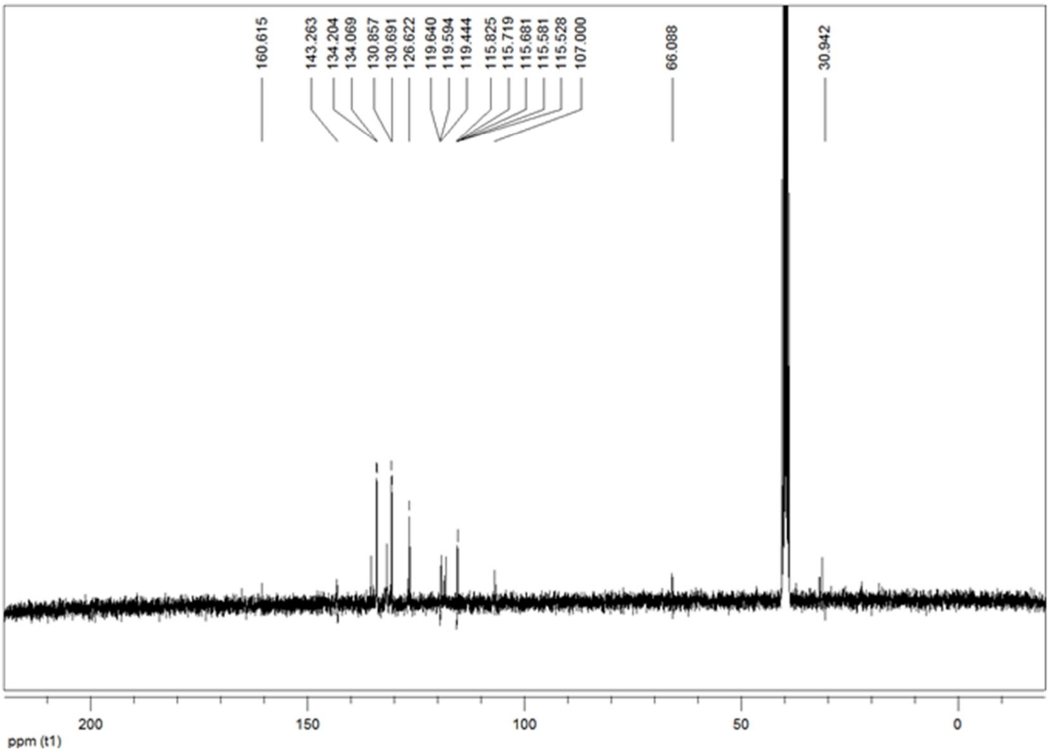
160.615
143.263
134.204
134.069
130.857
130.691
126.622
119.640
119.594
119.444
115.825
115.719
115.681
115.581
115.528
107.000
66.088
30.942
200 150 100 50 0
ppm (t1)

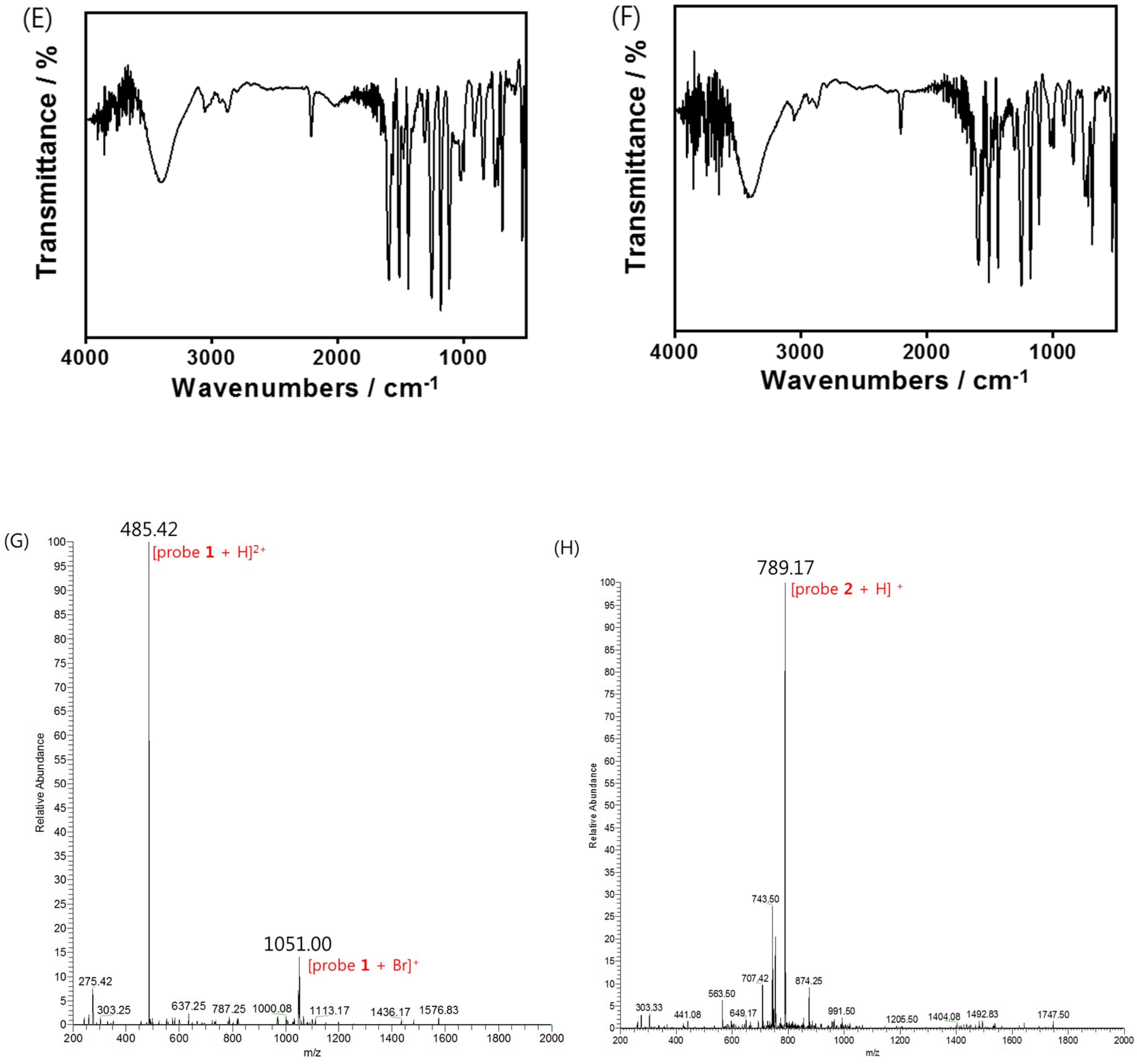

Figure S1. ^{1}H NMR spectra of probes (A) **1** and (C) **2** in DMSO – d_6. ^{13}C NMR spectra of probes (B) **1** and (D) **2** in DMSO – d_6. FT-IR spectra of probes (E) **1** and (F) **2**. ESI-MS spectra of probes (G) **1** and (H) **2**.

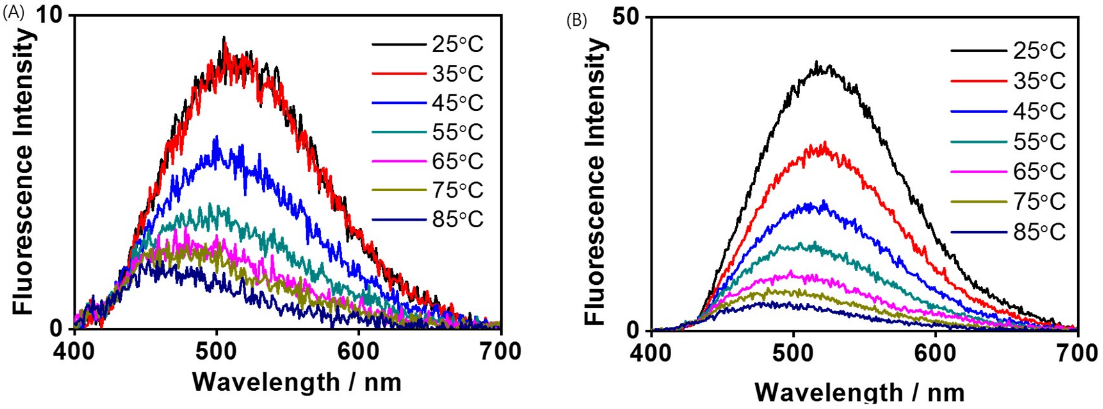

Figure S2. (A) Temperature dependent measurement of Fluorescence spectra of probe **1** (25 μM). (B) Temperature dependent measurement of Fluorescence spectra of probe **2**(6.25 μM).

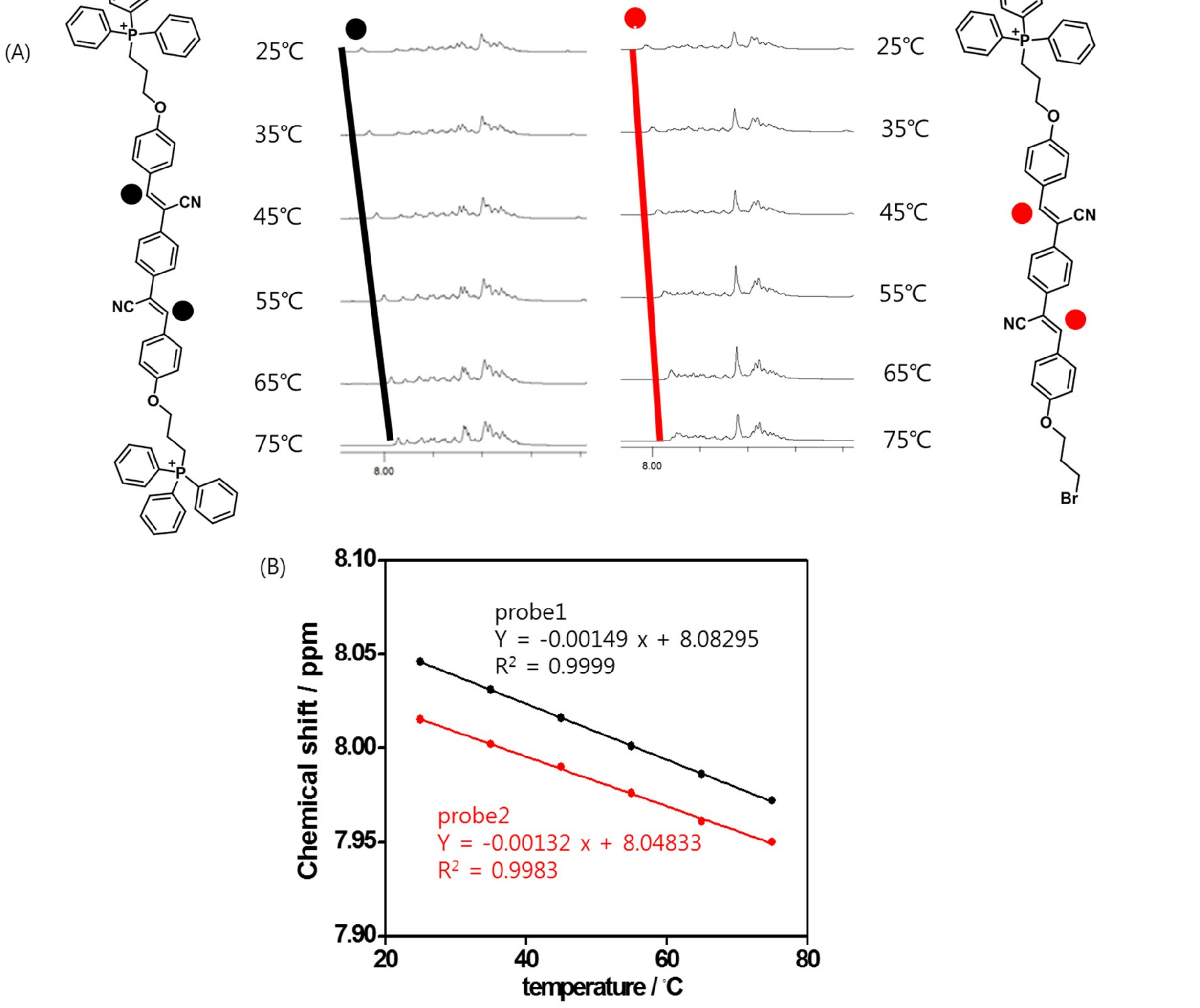

Figure S3. (A) Temperature dependent measurement of ^{1}H NMR spectra of probes **1** and **2** in DMSO-d_6/D_2O (99:1 v/v%). (B) Linear equation of chemical shift according to temperature.

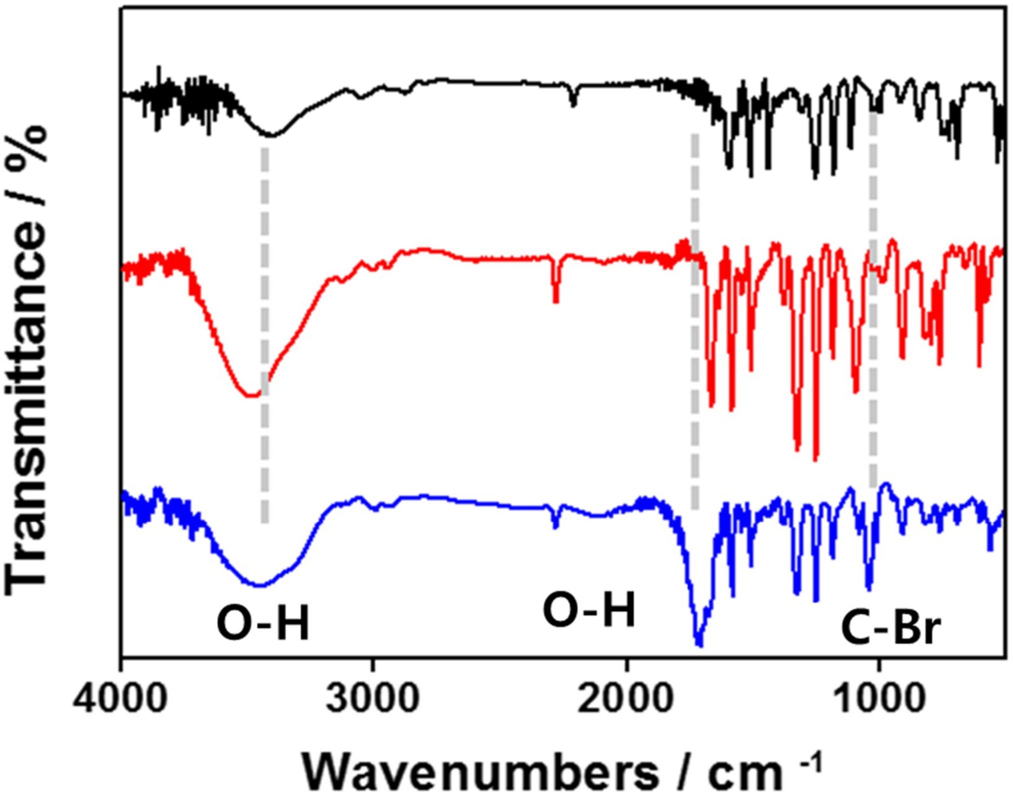

Figure S4. IR spectra of Probe **2** (100 μM) in DMSO/H_2O(1/99 v/v%). Black line is Probe **2**, Red line is Probe **2** in DMSO/H_2O (1/99 v/v%) solution, Blue line is Probe **2** with ClO^- (10 equiv.) in DMSO/H_2O(1/99 v/v%) solution.

.

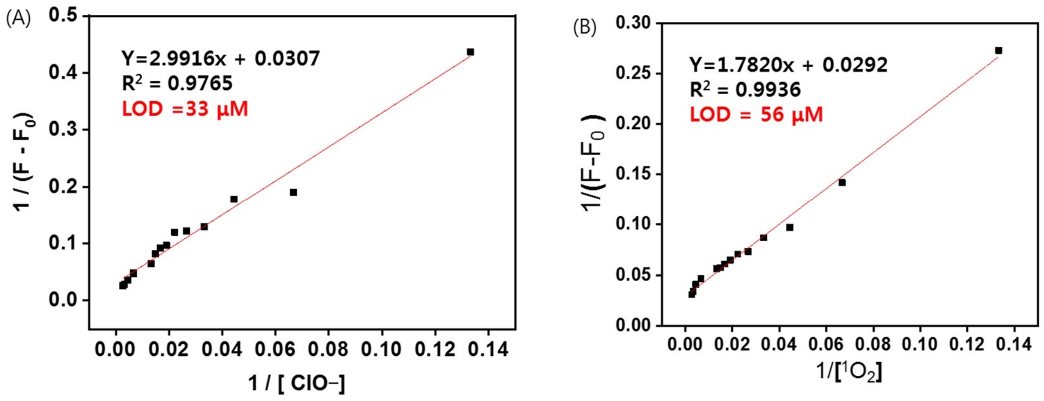

Figure S5. Fit linear equation of fluorescence intensity of probe **1** over (A) ClO^- and (B) $^1O_2$ to obtain limit of detection.

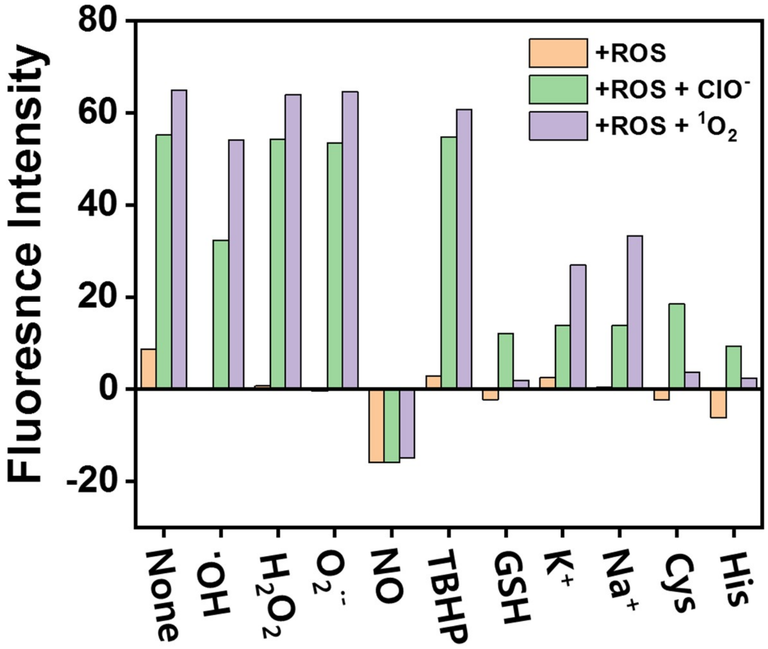

Figure S6. Competition-based fluorescence emission of probe **1** (25 μM) in the presence of various ROS (10 equiv.), Ions (10 equiv.) and Amino acids (10 equiv.) in DMSO/H_2O(1/99 v/v%).

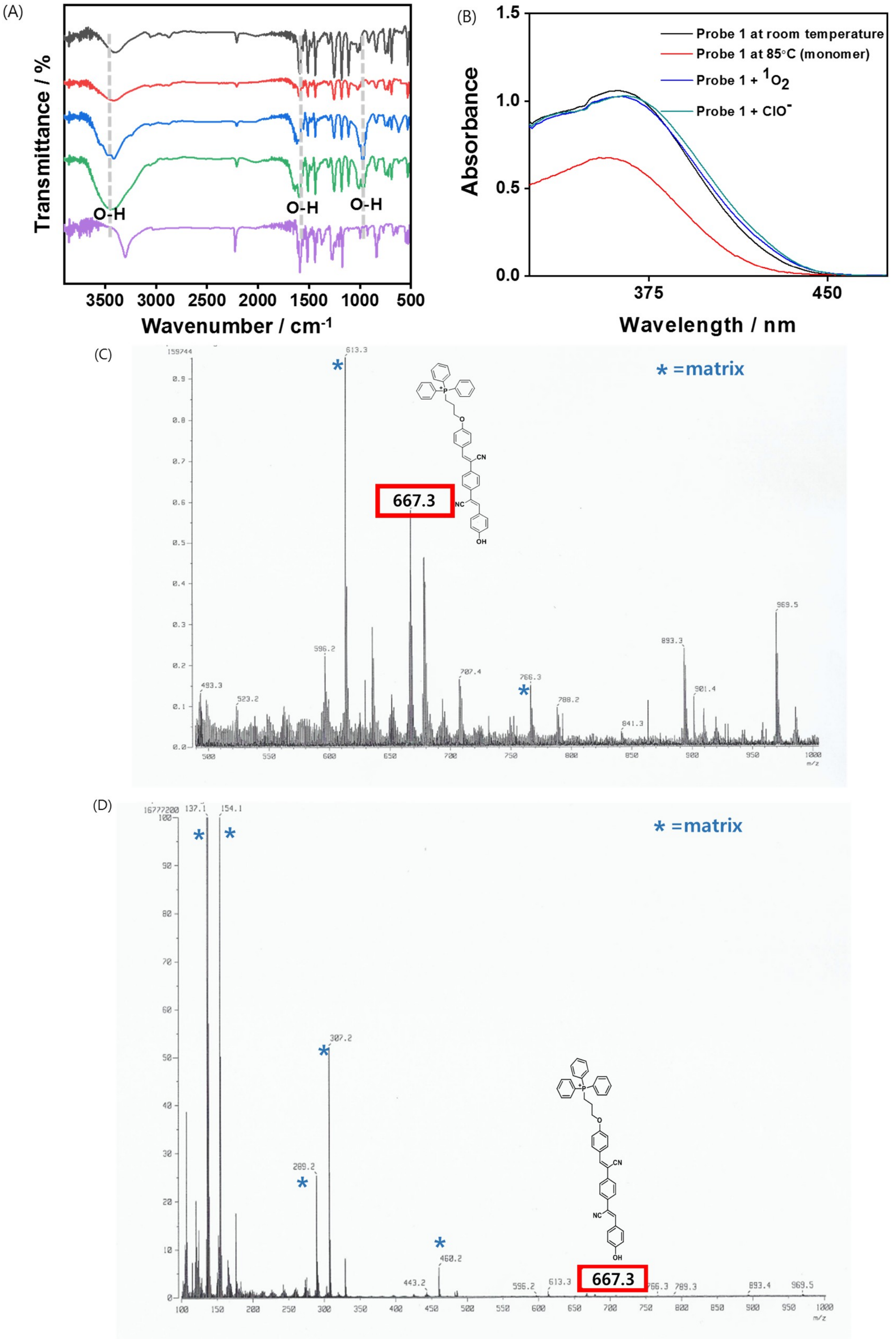

Figure S7. (A) IR spectra of **1** (100 μM) in DMSO/H_2O (1/99 v/v%). Black line is pristine probe **1**, Red line is freeze-dried self-assembled probe **1** from DMSO/H_2O (1/99 v/v%) solution, Blue line is self-assembled probe **1** with ClO^- in DMSO/H_2O(1/99 v/v%) solution, Green line is self-assembled probe **1** with $^1O_2$ in DMSO/H_2O (1/99 v/v%) solution and Purple line is dicyanostilbene (**1-Ref**). (B) UV- Vis spectra of probe **1** (100 μM) in DMSO/H_2O (1/99 v/v%) containing its monomer, self-assembled state, with $^1O_2$ or ClO^-. FAB-MASS spectra of probe **1** (1mM) after adding (C) ClO^- and (D) $^1O_2$ at NBA matrix.

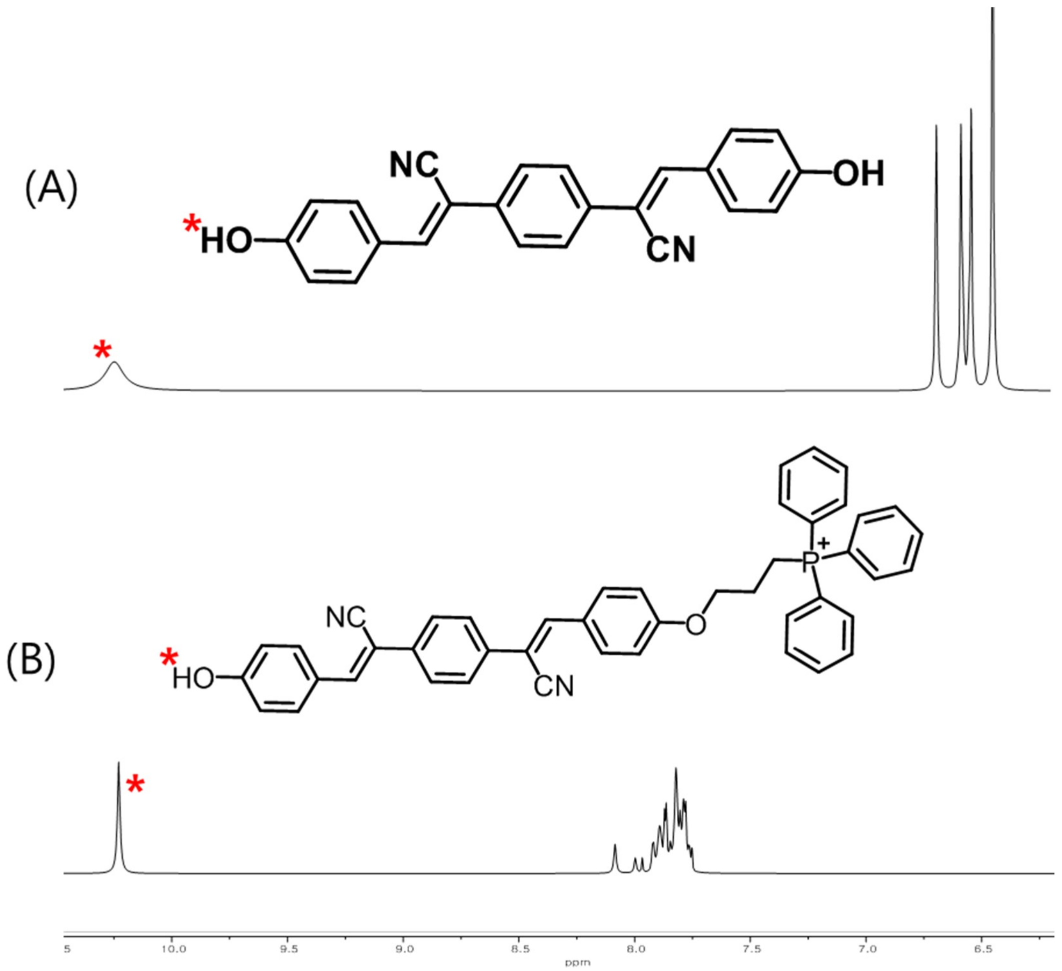

Figure S8. ^{1}H NMR spectra of (A) dicyanosilbene involving –OH group (**1-Ref**) and (B) probe **1** after addition of $^{1}O_2$.

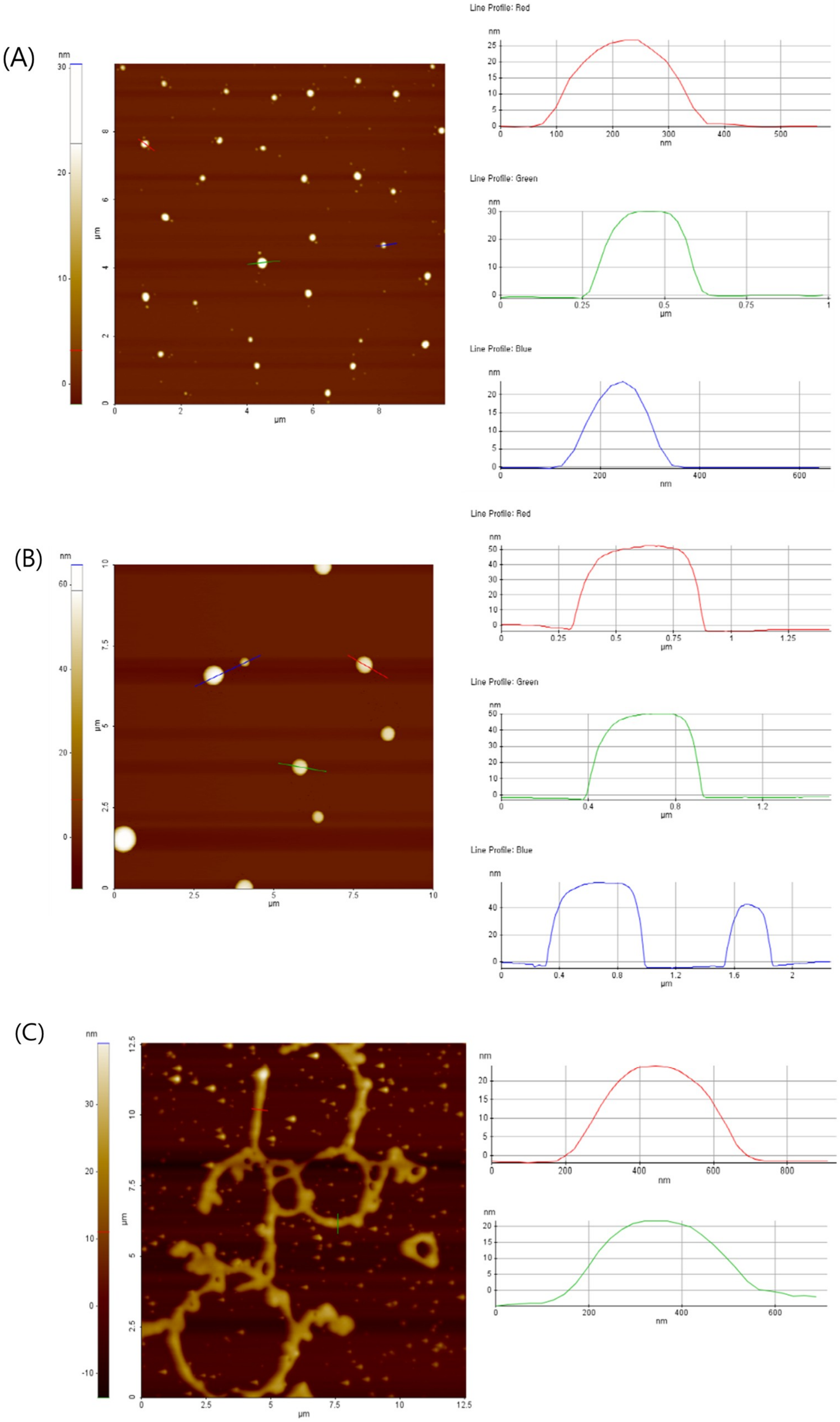

Figure S9. AFM images and height profile of (A) probe **1**, (B) probe **2** and (C) probe **1** + ClO^- .

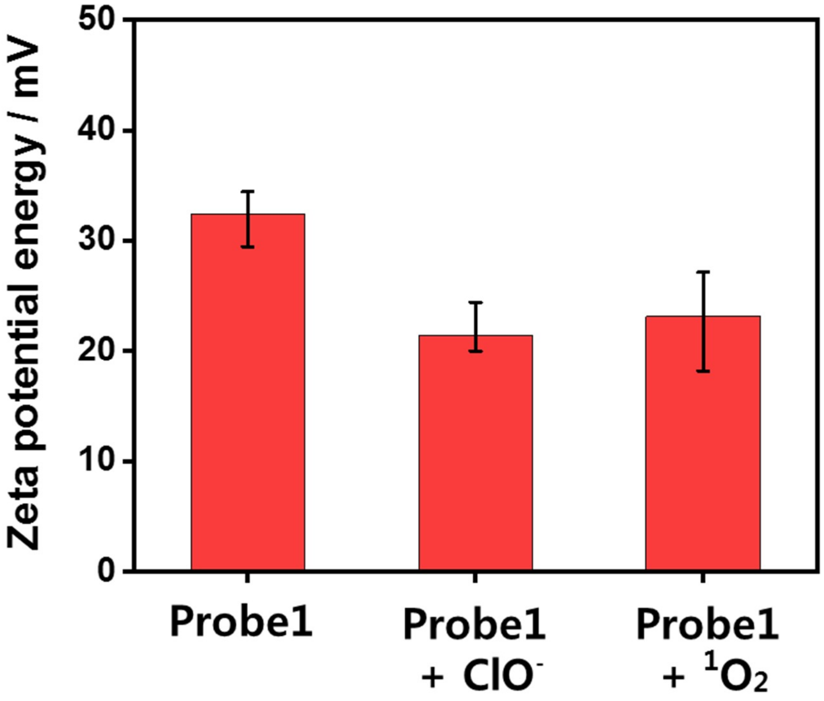

Figure S10. Zeta potential of probe **1** (none, addition of ClO^- , $^1O_2$) at 25 ℃.

Table S1. Characterization of probes **1** and **2**.

	Probe 1	Probe 2
Chemical Formula	$C_{66}H_{56}N_2O_2P_2^{2+}$	$C_{49}H_{45}BrN_2O_2P^+$
Molecular Weight	971.13 g/mol	804.79 g/mol
λ_{max} (monomer)	366nm	367nm
λ_{max} (aggregation)	371nm	372nm
Aggregation type	J-aggregation	J-aggregation
λ_{max} (Fluorecence)	512nm	512nm

References

S1. Artinez-Abadia, M.; Varghese, S.; Gimenez, R.; Ros, M. B. Multiresponsive luminescent dicyanodistyrylbenzenes and their photochemistry in solution and in bulk. *J. Mater. Chem. C* **2016**, *4*, 2886-2893.

S2. Niu, J.; Fan, J.; Wang, X.; Xiao, Y.; Xie, X.; Jiao, X.; Sun, C.; Tang, B. Simultaneous fluorescence and chemiluminescence turned on by aggregation-induced emission for real-time monitoring of endogenous superoxide anion in live cells. *Anal. Chem. (Washington, DC, U. S.)* **2017**, *89*, 7210-7215; Long, G. L.; Winefordner, J. D. Limit of detection. A closer look at the IUPAC definition. *Anal. Chem.* **1983**, *55*, 712A-724A.; Mun, G.; Jung, S. H.; Ahn, A.; Lee, S. S.; Choi, M. Y.; Kim, D. H.; Kim, J.-Y.; Jung, J. H. Fluorescence imaging for Fe^{3+} in Arabidopsis by using simple naphthalene-based ligands. *RSC Adv.* **2016**, *6*, 53912-53918.
